# Avoidance of Negative Emotional Contrasts as a Diagnostic Feature of OCD: A Receiver-Operator Characteristic Curve Analysis of the Contrast Avoidance Questionnaires

**DOI:** 10.1192/j.eurpsy.2024.736

**Published:** 2024-08-27

**Authors:** V. Swisher, M. G. Newman

**Affiliations:** ^1^Psychology, The Pennsylvania State University, State College, United States

## Abstract

**Introduction:**

The Contrast Avoidance Model (CAM) was developed to explain pathological worry in generalized anxiety disorder (GAD). The CAM posits that those with GAD are sensitive to sharp increases in emotions, and use worry to maintain heightened states of negative arousal to avoid these emotional shifts. Research has widely supported the CAM in the conceptualization of GAD, and has extended these findings to other disorders, including major depressive disorder (MDD) and social anxiety disorder (SAD). Despite the utility of the CAM model in informing the etiology of these conditions, research has yet to expand these findings beyond GAD, MDD, and SAD. Specifically, obsessive-compulsive disorder (OCD), which co-occurs with GAD, MDD, and SAD in adults at a rate of 15.0%, 40.7%, and 14.7%, respectively, and shares many of their etiological features, has yet to examined in the context of the CAM. Thus, examining CA as a relevant mechanism and therapeutic target for OCD is an unstudied conceptual framework that may offer meaningful clinical utility.

**Objectives:**

The present study used receiver operator curve (ROC) analyses to examine the predictive utility of the CAQ-W and CAQ-GE in detecting probable OCD in a large undergraduate sample. We hypothesized that the CAQ-W and CAQ-GE would be higher in participants with probable OCD and would offer sufficient sensitivity and specificity in predicting probable OCD.

**Methods:**

1259 undergraduates were recruited for a mass University screening. Participants were included in the OCD group (N = 291) if they met diagnostic criteria for OCD (DOCS total score > 20). Participants were included in the nondisordered group (n = 249) if they did not meet diagnostic criteria for any of the screened disorders (SAD, MDD, GAD, OCD, panic disorder, post-traumatic stress disorder, OCD, borderline personality disorder), denied suicidality, and denied receiving mental health treatment in the last 12 months. ROC analyses were used to examine the accuracy of the CAQ-W and the CAQ-GE in detecting probable OCD.

**Results:**

Results of ROC analyses are reported in Table 1. AUC values for the CAQ-W and CAQ-GE were significantly different from the null hypothesis (AUC = .50, p < .001), and demonstrated excellent (.89) to outstanding (.91) accuracy in predicting probable OCD (Figure 1), respectively. Optimal sensitivity and specificity to detect probable OCD (Table 2) was achieved at a cut off score of 67.5 for the CAQ-W (Sensitivity = 81.4%; Specificity = 82.3%) and a cutoff score of 43.5 for the CAQ-GE (Sensitivity = 84.9%; Specificity = 85.5%).

**Image:**

**Figure:**
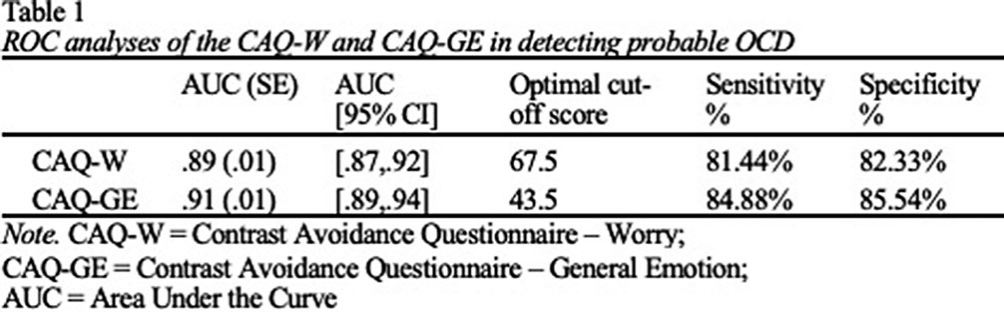

**Image 2:**

**Figure:**
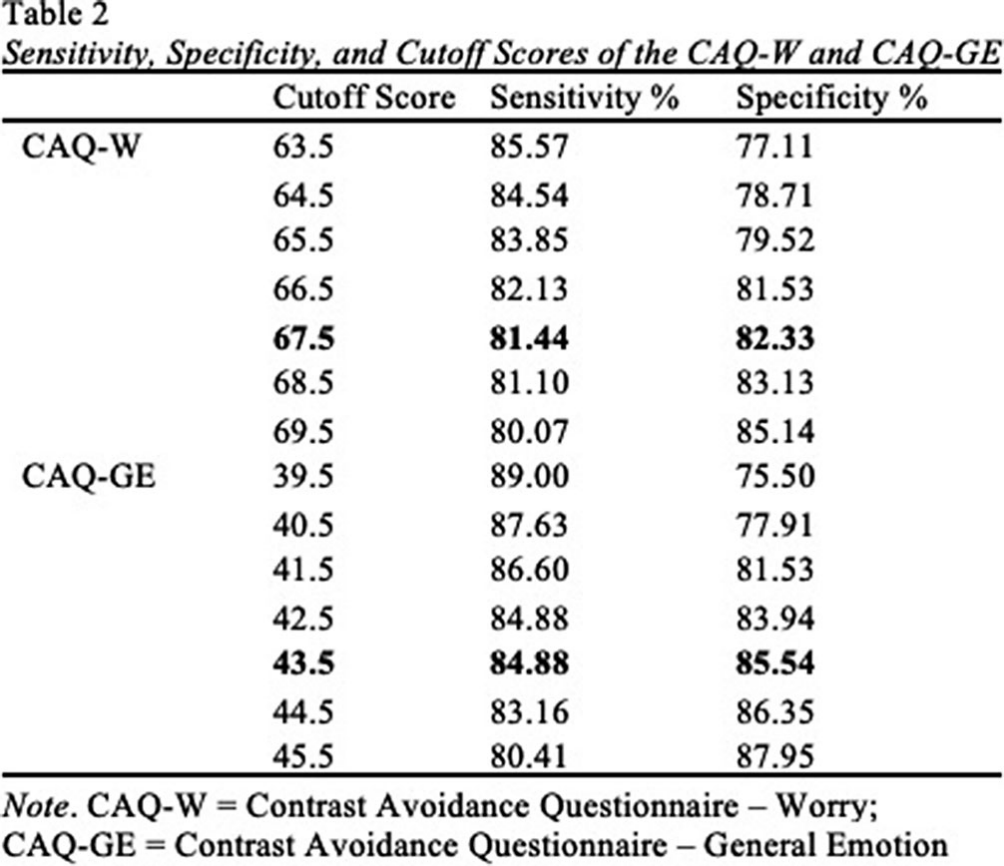

**Image 3:**

**Figure:**
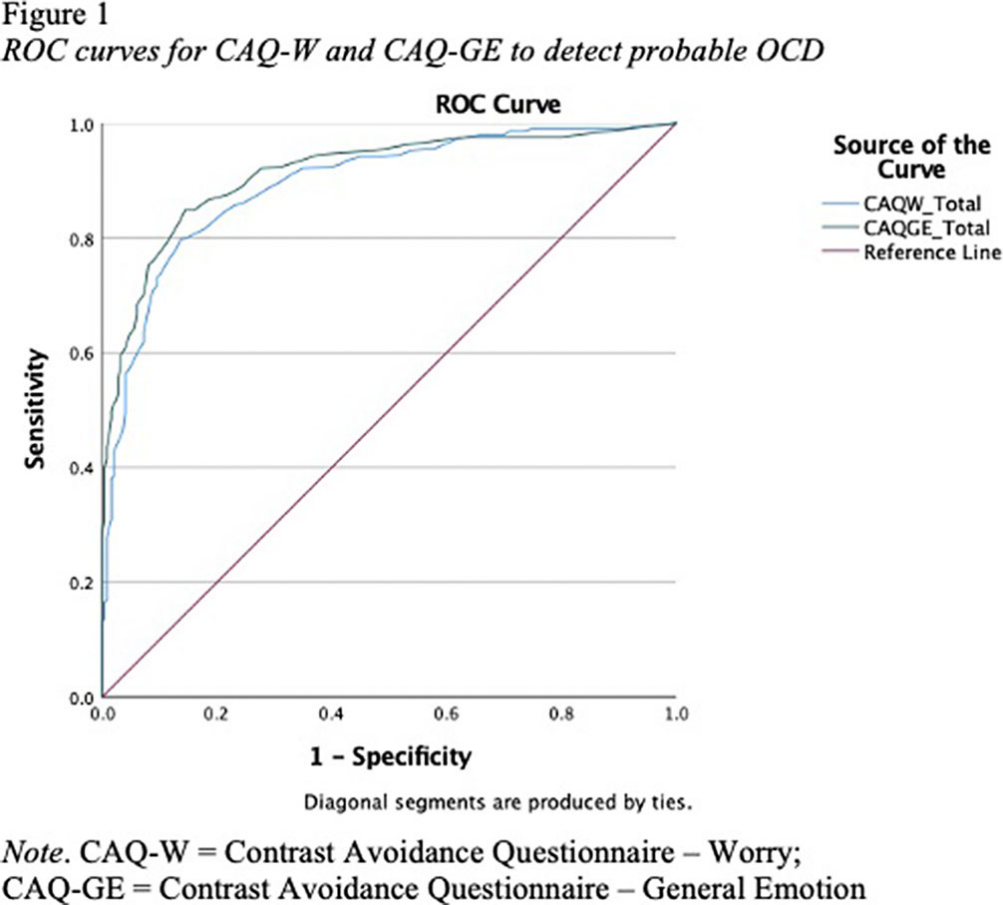

**Conclusions:**

Results suggest that OCD can be accurately characterized by CA. Findings also highlight the utility of examining CA as a relevant maintenance factor for OCD symptoms. Future research should examine the impact of CA on OCD symptoms in-laboratory and ecological settings.

**Disclosure of Interest:**

None Declared

